# Skin tattooing as an effective tool for delivering DNA and protein vaccine immunogens

**DOI:** 10.1186/1742-4690-9-S2-P338

**Published:** 2012-09-13

**Authors:** Y Chiu, X Jiang, R Kumar, CE Hioe, S Zolla-Pazner, X Kong

**Affiliations:** 1 NYU School of Medicine, New York, NY, USA; 2 Veterans Affairs New York Harbor Healthcare System, New York, NY, USA; 3 Departments of Pathology / NYU School of Medicine, New York, NY, USA

## Background

Skin is an excellent site for vaccine administration due to the abundance of antigen presenting cells and easy access. When DNA or protein is delivered into the skin, uptake and expression of immunogens can lead to a rapid and robust induction of immune responses.Skin tattooing has been suggested as a useful tool delivering vaccines intradermally. We have chosen to use the skin tattooing technique to deliver both DNA and protein immunogens that are focusing the immune response on the highly immunogenic V3 region of HIV-1 gp120.

## Methods

A GFP-containing plasmid was used first to optimize the protocol for skin tattooing on BALB/c mice. Subsequently, BALB/c mice were immunized using a DNA prime/Protein boost regimen. DNA skin tattooing was used first as the prime to deliver a gp120-encoding DNA plasmid intradermally at weeks 0, 2, and 4. The animals were then given a fusion protein, V3-bearing cholera toxin subunit B (V3-CTB), via skin tattooing (Group1; n=5) or intramuscular injection (Group2; n=5) at weeks 10 and 14. Two weeks after the final immunization, the sera were collected and analyzed.

## Results

GFP expressed in skin cells was visualized by confocal microscopy showing that skin tattooing can effectively deliver DNA into these cells. ELISA assays using sera drawn after the final immunization confirmed that tattoo-delivered DNA followed by V3-CTBelicited V3 binding antibodies, and these antibodies have potent neutralizing activities in an HIV pseudovirus neutralization assay.

## Conclusion

Immunization by skin tattooing is an easy, effective, and economical way to administer both DNA and protein vaccines. Interestingly, the needle perforations may serve as a potent and natural adjuvant for the immunization, making skin tattooing a promising vaccine delivery technique.

